# Repetition suppression to objects is modulated by stimulus-specific expectations

**DOI:** 10.1038/s41598-017-09374-z

**Published:** 2017-08-18

**Authors:** Christian Utzerath, Elexa St. John-Saaltink, Jan Buitelaar, Floris P. de Lange

**Affiliations:** 1 Radboudumc, Donders Institute for Brain, Cognition, and Behaviour, Department of Cognitive Neuroscience, Geert Groteplein-Noord 21, 6525 EZ Nijmegen, The Netherlands; 2 Radboud University, Donders Institute for Brain, Cognition and Behaviour, Kapittelweg 29, 6525 EN Nijmegen, The Netherlands

## Abstract

Repeated exposure to the same stimulus results in an attenuated brain response in cortical regions that are activated during the processing of that stimulus. This phenomenon, called repetition suppression (RS), has been shown to be modulated by expectation. Typically, this is achieved by varying the probability of stimulus repetitions (P_rep_) between blocks of an experiment, generating an abstract expectation that ‘things will repeat’. Here, we examined whether stimulus-specific expectations also modulate RS. We designed a task where expectation and repetition are manipulated independently, using stimulus-specific expectations. We investigated to which extent such stimulus-specific expectations modulated the visual evoked response to objects in lateral occipital cortex (LOC) and primary visual cortex (V1), using functional magnetic resonance imaging (fMRI). In LOC, we found that RS interacted with expectation, such that repetition suppression was more pronounced for unexpected relative to expected stimuli. Additionally, we found that the response of stimulus-preferring voxels in V1 was generally decreased when stimuli were expected. These results suggest that stimulus-specific expectations about objects modulate LOC and propagate back to the earliest cortical station processing visual input.

## Introduction

Repeated exposure to the same stimulus results in a weaker brain response in cortical regions that are relevant for processing that stimulus^1–3^. This phenomenon, called repetition suppression (RS), has been widely studied within different modalities and brain regions. Despite this, its underlying mechanisms are still not fully understood^4^. This may be explained by the fact that RS is multifaceted and the result of several concurrent processes^5^.

One potentially important modulatory factor of RS is stimulus expectation. In an elegant fMRI experiment, Summerfield and colleagues showed that RS in the fusiform face area (FFA) was more pronounced when repetitions of face stimuli were expected relative to unexpected^6^. In spite of some controversy regarding the generalizability and interpretation of this observation^7, 8^, later studies have consistently observed a modulation of RS by expectation for faces in face-selective visual regions^9^, and for familiar non-face stimuli in the lateral occipital complex (LOC)^10^.

These previous studies investigated modulations of RS by making repetitions more or less frequent: i.e., by varying P_rep_, or the probability of a repetition, participants come to generally (not) expect repetitions. In life however, expectations often pertain to specific stimuli (e.g., expecting to see a cat upon hearing a meow). Whether and how RS is modulated by such stimulus-specific expectations is unclear. Although, there are several neurophysiological studies showing a modulation of neural activity by stimulus-specific expectations^11–13^, To our knowledge, no one has yet demonstrated the modulation of RS by such stimulus-specific expectations.

**Figure 1:**
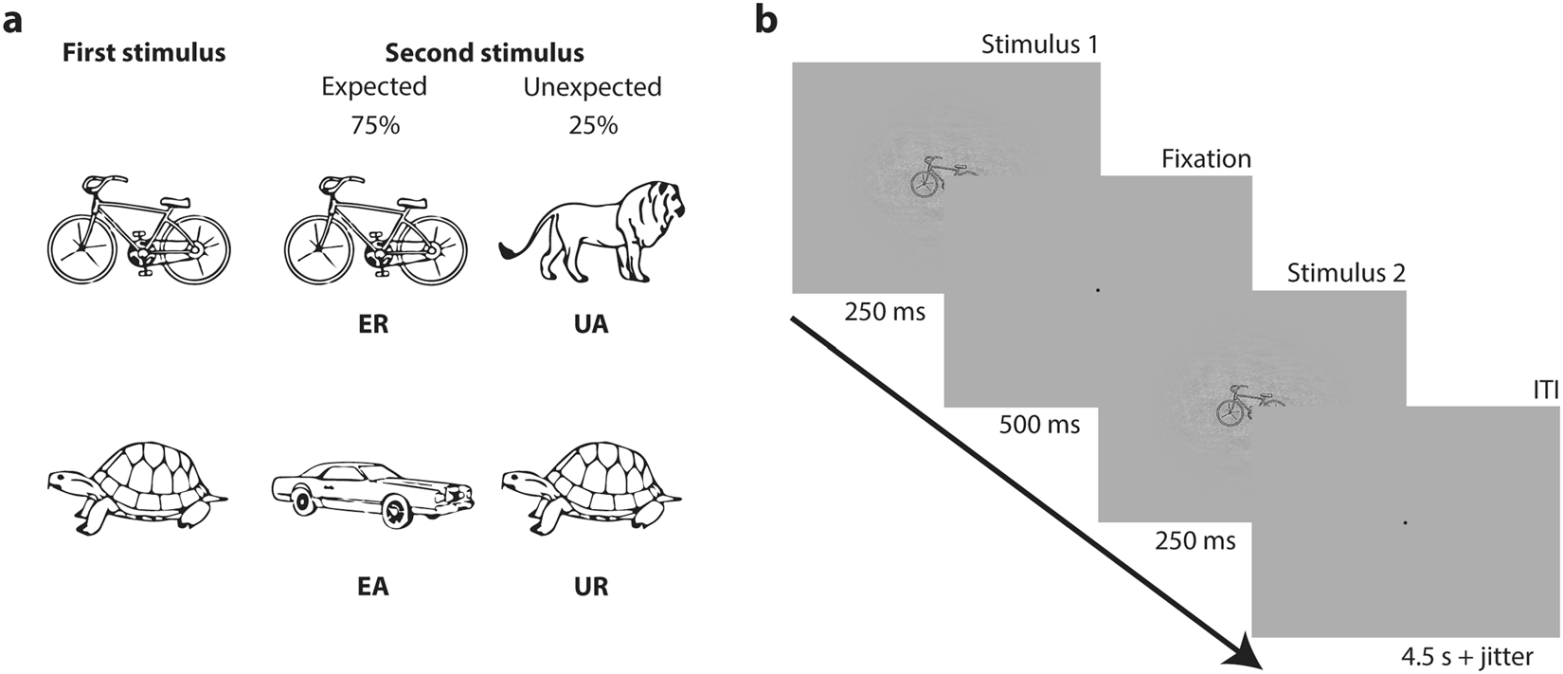
Paradigm and task. **(a)** Examples of fixed stimulus pairings. Participants learned that some stimuli are most likely to repeat, whereas others are most likely to alternate, thus creating expected repetitions (ER) and expected alternations (EA), as well as unexpected repetitions (UR) and unexpected alternations (UA). **(b)** Stimulus display, here showing an expected repetition (ER) trial. In the behavioral discrimination task, participants responded to the category of the second stimulus (vehicle or animal) during the inter-trial interval (ITI). During the fMRI oddball task, participants responded to occasional oddball targets (17.4% of trials) in which the stimulus was shown at 60% of its normal size.

Here, we extend the literature by investigating how stimulus-specific expectations about objects modulate the response in object-selective cortical area LOC, using fMRI. We addressed this question by presenting our participants with different objects while varying stimulus expectation and stimulus repetition independently (Fig. 1a). Our participants learned that that some stimuli were likely to repeat, whereas other stimuli were likely to alternate to a specific different stimulus. We then tested the effects of these expectations by occasionally violating them during a behavioral category discrimination task, and a subsequent fMRI experiment.

Additionally, it is an open question whether these modulations of RS by expectation, which are typically observed in higher-order visual regions such as LOC, propagate back to primary visual cortex (V1). Many neurocomputational models describe visual perception as a generative process in which prediction signals from upstream brain areas propagate down to modulate sensory processing at the earliest levels including V1^14–17^. However, studies on the expectation modulation of RS so far do not report whether their reported effects propagate back to V1. To explore this issue, we expanded our analysis to primary visual cortex. If expectations about object repetitions were to propagate along the visual hierarchy, we hypothesized to find corresponding expectation effects in LOC and V1.

Finally, a tangential goal of our study was to determine whether expectation and repetition effects would be modulated by autistic personality traits. This question was inspired by current accounts that cast autism as a disorder whereby the integration of priors and sensory evidence is altered^18–20^; recent work saw a modulation of repetition but not expectation suppression (ES) by normal variation in autistic traits^21, 22^.

To preview our results, we found that stimulus-specific object expectations modulated RS in LOC. This modulation propagated down to V1, where voxels decreased their response when stimuli were expected. These results were unmodulated by normal variation in autistic personality traits in our sample.

## Methods

### Participants

We recruited 24 right-handed, healthy student participants (17 female, mean age 22.8 ± 2.7 years) who gave written, informed consent and received course credit for their participation. Experimental procedures were approved by the local ethics committee (Commissie Mensgebonden Onderzoek Regio Arnhem-Nijmegen, the Netherlands) under the general ethics approval (“Imaging Human Cognition”, CMO 2014/288) and the experiment was conducted in compliance with these guidelines.

### Stimuli

We created stimuli (a lion, turtle, bike, and a car; Fig. 1a) as object outlines following designs by Rossion and Pourtoise^23^. Using the SHINE toolbox^24^, we matched these images on their spatial frequencies and mean luminance. We also generated a Fourier scrambled version of each stimulus, by randomly shuffling the phase of its spatial frequencies. The scrambled images were again matched in terms of spatial frequency and luminance using the same SHINE procedure. Stimuli subtended a visual angle of approximately 7.5° by 5°. The tasks were programmed using MATLAB R2012b (The MathWorks, Natick, MA, USA) in combination with PsychToolbox^25^.

On each trial, we presented the participants with two consecutive stimuli (a pair; Fig. 1b). Pairs consisted of either the repetition of a single stimulus, or an alternation between two stimuli. There were four different stimuli, of which two had a 75% probability of repeating and two had a 75% probability of alternating, resulting in four possible outcomes: expected and unexpected repetitions, as well as expected and unexpected alternations (Fig. 1a). Stimulus transitions would always occur across category (e.g., from animal to vehicle). Which stimuli would repeat was counter-balanced between participants. Trial sequences were presented in randomized order.

### General procedure and tasks

Within five days preceding the experiment, participants were trained on two tasks. First, participants practiced 128 trials of a category discrimination task. In this task, participants indicated on each trial whether the second stimulus in each pair depicted an animal or a vehicle (see Fig. 1b). Only expected pairs were shown to facilitate learning of which stimuli would repeat or alternate. All trials started with the sequential presentation of one of the stimulus pairs. Each stimulus was shown for 250 ms, separated by an inter-stimulus interval of 500 ms. Trials ended with an inter-trial interval of 4.5 s, to which up to 2 s of jitter were added (Fig. 1b).

**Figure 2:**
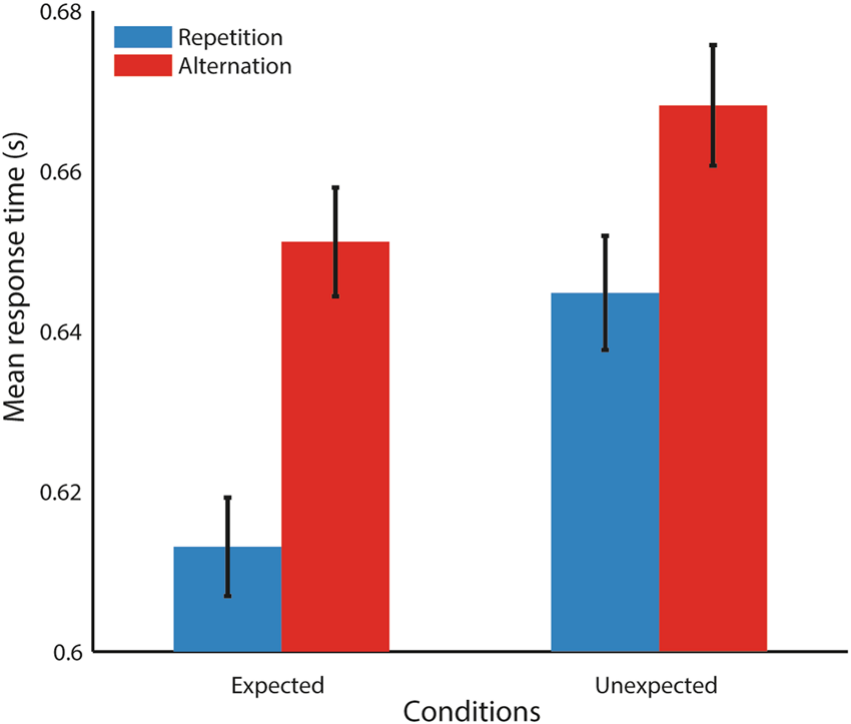
Reaction times during category discrimination task (preceding the fMRI experiment). Error bars reflect within-subject standard error of the mean (SEM)^46, 47^. There were significant main effects of repetition and expectation. Additional information about responses can be found in supplemental Figure S3.

During the subsequent testing session, all participants completed both a behavioral and fMRI experiment. Just prior to the fMRI experiment, participants performed 96 trials of the behavioral category discrimination task. Now, expected repetitions (ER) and expected alternations (EA) occurred on 75% of the trials, such that they could be contrasted with unexpected repetitions (UR) and unexpected alternations (UA). The reaction time measurements collected during the behavioral task allowed us to probe whether expectation and repetition affected the behavioral response to the stimuli, and whether participants had learned the transition probabilities (cf. Fig. 2). Aside from measuring these behavioral effects, this procedure ensured that participants were amply exposed to the trained stimulus transitions.

Afterwards, participants performed an oddball task in the fMRI. During this task, occasionally the second stimulus of a pair was presented at 60% of its original size (the target), and participants pressed a button whenever they detected a target. Of note, the learnt transition probabilities were irrelevant for this task. Of all trials, 17.4% contained an oddball target, and target presentation was counterbalanced across conditions. Target trials were not considered for fMRI analysis. This means that any sort of behavioral response could not confound the fMRI responses of the participants and further ensured people attended all conditions equally. Finally, 13% of trials were null events in which no stimuli were presented. These null trials allowed us to measure the baseline response of the brain against which the experimental conditions could be contrasted, and served to de-correlate the other conditions in which visual stimuli were presented^26^.

During fMRI, participants engaged in four runs of the oddball task for a total of 368 trials during approximately 40 minutes total scanning time. Lastly, we performed a localizer scan to identify object-sensitive brain regions. The localizer lasted approximately 16 minutes and consisted of blocks of 10 s, during which one stimulus or scrambled image was shown at a time, flashing on and off at 2 Hz. Participants’ task was to detect whenever a stimulus was presented slightly off-centre for about 300 ms, which occurred approximately twice per block.

To quantify autistic traits, participants also completed the Autism Spectrum Quotient questionnaire, a 50-item, non-clinical instrument that measures personality traits related to the autism spectrum^27^.

### Image acquisition and pre-processing

Images were acquired on a 1.5 T Siemens Magnetom Avanto MRI system (Siemens, Erlangen, Germany). A high-resolution structural image was created using a T1-weighted sequence (TR = 2.25 s, TE = 2.95 ms, 1 × 1 × 1 mm in-plane resolution). Functional images were acquired using a 2D EPI sequence (TR = 2.02 s, TE = 40 ms, 3 × 3 × 3.5 mm, 26 sagittal slices). Data were pre-processed using SPM8 (Wellcome Trust Centre for Neuroimaging, London, UK). We discarded the first four volumes of every run in order to allow for initial equilibrium. Functional images were first spatially realigned to the mean and then corrected for slice timing. The mean functional image was brought in register with the T1. The T1 was furthermore segmented using SPM8’s segment function, which yielded normalization parameters into MNI space. Finally, functional images were normalized into MNI space, and smoothed (6 × 6 × 6 mm FWHM).

### Construction of individual Regions Of Interest (ROIs)

**Figure 3:**
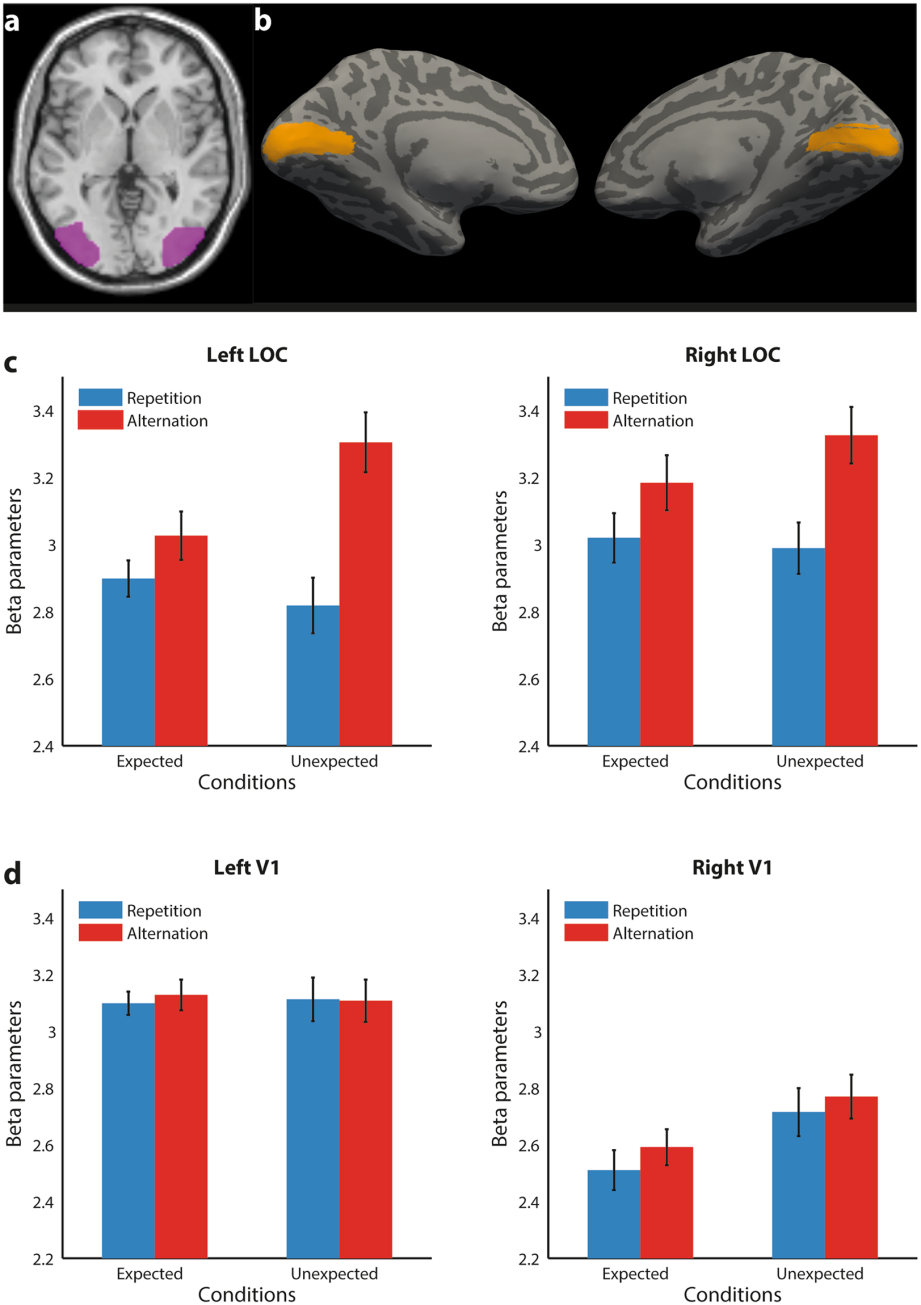
fMRI ROI results. **(a)** LOC ROI. Panel shows in violet the group response to the object > scrambles contrast during the localizer, and superimposed on a transversal slice of SPM’s MNI template at z = 0. For each participant individually, per hemisphere the 100 most object-selective voxels were chosen within this anatomical site. **(b)** Reconstructed V1 (left, right) shown in yellow on an inflated brain from one representative participant. **(c-d)** Mean parameter estimates for LOC and right V1 per condition. Error bars reflect within-subject SEM. Additional information on the ROI and beta parameters within these ROIs can be found in the supplementary Figures S1, S2, and S5.

For each participant, we defined two participant-specific Regions Of Interest (ROIs): V1 and LOC. LOC was individually identified based on each participant’s LOC localizer. We identified the 100 voxels per hemisphere that responded most strongly to objects in comparison to scrambled objects during the localizer session, based on the corresponding contrast image. To ensure reasonably consistent anatomical location between subjects, only voxels were considered that were also part of a significant cluster for objects > scrambles at the group level, at a voxel threshold of *p* < 0.001 (Fig. 3a). Voxels that were located near V1 were also not considered, to prevent potential overlap between ROIs (see below).

In order to identify every participant’s V1, we used Freesurfer’s automatic anatomical reconstruction algorithm to parcellate every participant’s T1 in native space (surfer.nmr.mgh.harvard.edu/)^28^. We used an automated method to predict V1 based on cortical folds. This method can be used to predict the retinotopic organization of striate cortex for an individual with accuracy equivalent to 10–25 min of functional mapping^28, 29^ (Fig. 3b; Figure S1). The reconstructed surface was brought in register with the functional scans and the labels corresponding to the predicted sites of V1 were converted into volume space. Using SPM8, this ROI was transformed into MNI space. To ensure the selection of voxels with a positive response to objects, we only considered voxels that had a positive response to the contrasts objects > scramble and objects > baseline. For data analysis, we then selected a subset of the 100 most responsive voxels to the contrast objects > baseline in V1. This method ensured that these voxels were responsive to the stimuli and located at anatomically plausible sites. Additional analysis after data acquisition showed that both LOC and V1 had consistent condition means across different potential ROI sizes (Figure S2). Finally, any voxels that, based on this selection procedure, came into consideration for both V1 and LOC were discarded entirely. This ensured that there was no overlap between the ROIs.

### Statistical analysis

Reaction times during the behavioral experiment and the oddball trials during the fMRI experiment were analyzed using a 2 (expected versus unexpected) × 2 (repetition versus alternation) analysis of variance (ANOVA).

With regards to the fMRI experiment, our ROI analysis was based on a General Linear Model (GLM) performed in SPM8. We used a 128 s high-pass filter to remove scanner drifts. For the main experiment, we modeled separate regressors for expected repetitions, unexpected repetitions, expected alternations, unexpected alternations, oddballs, and null events. For the localizer, we modeled null events, object and scramble presentations. These regressors were then convolved with SPM8’s canonical hemodynamic response function. We furthermore included the motion parameters obtained during realignment, as well as their first and squared first derivatives as nuisance regressors.

For the ROI analysis, we then took the resulting beta parameter estimates within LOC and V1 and calculated each participant’s mean parameter estimate per ROI, hemisphere, and condition. For each region, we subjected these to a 2 (expected versus unexpected) × 2 (repetition versus alternation) × 2 (left versus right hemisphere) within-subjects repeated measure analysis of variance (ANOVA). Interactions were further examined using post-hoc t-tests. We also gauged potential modulatory effects of autistic personality traits on RS and ES using an analysis of covariance, whereby mean-centered AQ was entered as a covariate for the factors expectation, repetition, and hemisphere.

## Results

### Behavioral performance during the category discrimination experiment

Participants were highly accurate (95% ± 8%, mean ± SD) during the behavioral category discrimination task experiment, indicating that participants successfully engaged in the task. Responses were faster when the second stimulus was a repetition compared to an alternation (*F*(1,23) = 12.62, *p* = 0.002), and when the second stimulus was expected compared to unexpected (*F*(1,23) = 21.07, *p* = 1.3e-4, see Fig. 2). There was no interaction between these two factors (*F*(1,23) = 1.33, *p* = 0.26). Thus, behavioral performance in the discrimination task was sensitive to both stimulus repetition and stimulus expectation. Additional analysis showed that effects of expectation and repetition on response time emerged already at the start of the experiment and were stable throughout, indicating that participants fully learned the stimulus transition prior to the fMRI experiment (Figure S3).

### Behavioral performance during the fMRI oddball experiment

During the fMRI experiment’s oddball task, participants were highly accurate at detecting the oddball trials during the fMRI session (97% ± 8%, mean ± SD), indicating that participants successfully engaged in the task. Neither percentage correct nor reaction times were affected by expectation and repetition during the fMRI oddball task (see Figure S4).

### Effects of stimulus repetition and expectation in LOC and V1

First, we examined the effects of repetition and expectation in LOC. In line with previous reports of repetition suppression for objects in this region, repetitions evoked less activity than alternations (*F*(1,23) = 11.72, *p* = 0.0023; see Fig. 3c). Furthermore, there was a significant interaction between repetition and expectation (*F*(1,23) = 11.4, *p* = 0.0011). Post-hoc tests revealed that repetition suppression was significantly stronger for unexpected compared to expected stimuli (*t*(23) = 2.21, *p* = 0.038). These effects did not interact with hemisphere (all *p* > 0.45).

In V1, there was no main effect of repetition (*F*(1,23) = 0.55, *p* = 0.46) nor did repetition interact with hemisphere (*p* = 0.48). Furthermore, expectation did not significantly modulate the visual response (*F*(1,23) = 2.2, *p* = 0.15; Fig. 3d). However, expectation interacted significantly with hemisphere (*F*(1,23) = 5.22, *p* = 0.025). This prompted us to inspect early visual cortex separately for each hemisphere. While there were no effects in the left hemisphere (all *p* > 0.8), the right hemisphere showed significant expectation suppression (*F*(1,23) = 5.1, *p* = 0.034). The expectation x repetition interaction was not significant (*F*(1,23) = 0.03, *p* = 0.86).

Additional analysis showed that these results were qualitatively similar for different selections of the amount of voxels included for each ROI (see Figure S2). We furthermore probed the stability of the expectation effect over time by contrasting expected and unexpected trials separately for each of the four runs of the experiment. This analysis showed that the modulatory effects of expectation were stable over time, as they did not behave differently during the different runs of the task (Figure S5).

### Modulation of expectation and repetition effects by AQ

Our sample had an average AQ of 14.79 (SD = 7.99), falling within the range of expected scores for healthy individuals^27^. Neither in LOC (all *p* > 0.7) nor in V1 (all *p* > 0.54) did effects of expectation or repetition covary with AQ. The expectation x repetition interaction did not covary with AQ in either region (both *p* > 0.48).

## Discussion

Here we showed that stimulus-specific expectations about objects modulate repetition suppression (RS) in object-selective area LOC. This extends the literature on modulations of RS by expectation, which focused on a more general form of expectation by manipulating the overall probability that ‘things will repeat’. Furthermore, the expectation modulation propagated to regions within V1, resulting in activity suppression for expected stimuli.

### Interaction between stimulus expectation and stimulus repetition in LOC

We observed expectation modulations of RS for stimulus-specific expectations. Interestingly, our results – larger RS for unexpected stimuli – may appear different from those described earlier^6, 8^, where RS effects are reported to be boosted when repetitions are *expected*. This contradiction, however, is more apparent than real. In the papers by Summerfield *et al*. and Larsson and Smith, the difference in BOLD activity was assessed between alternations and repetitions (i.e., RS) in the context of *frequent* repetitions (rep block) or *infrequent* repetitions (alt block). When quantifying RS during blocks where repetitions are frequent, the researchers contrasted frequent (i.e., expected) repetitions with infrequent (i.e., unexpected) alternations. In our paradigm, this corresponds to expected repetitions and unexpected alternations. Conversely, in blocks where repetitions are infrequent, they compared frequent (i.e., expected) alternations with infrequent (i.e., unexpected) repetitions (see also ref. 30). When comparing the condition means to our corresponding conditions, the differences between conditions appear in good agreement.

Contrary to P_rep_ designs, the stimulus-specific design of the current study affords the possibility to create (potentially more precise) stimulus expectations, which can be manipulated independently of stimulus repetition between trials. This may provide some benefits over relative to P_rep_ manipulations.

### Stimulus familiarity may facilitate expectation effects in LOC

We find that the BOLD response in LOC to objects is modulated by expectation. Earlier studies that used non-face stimuli had been inconclusive: Kovács and colleagues for instance found no P_rep_ effects in ventral visual cortex for everyday objects^31^, congruent with single-unit recordings in monkeys^7^. However, in a subsequent fMRI study, expectation modulations of RS were found for non-face stimuli in ventral visual cortex. These modulations occurred for roman letters but not false fonts^10^. Supposedly, the familiarity that participants had with roman letters (but not false fonts) enabled expectation effects to occur^10^. Yet familiarity alone does not seem to suffice for eliciting expectation effects: when Kovács and colleagues^31^ presented their participants everyday objects, they observed no corresponding effect of expectations. These objects, too, must have at least been recognizably familiar to the participants. Why would familiar roman letters and our stimuli, but not (familiar) everyday object cause expectation effects?

A reconciliatory view would be that perhaps conceptual familiarity alone is not sufficient to effect these modulations. Instead, perceptual rather than conceptual familiarity might be required. Grotheer and Kovács^10^ themselves argue that many phenomena with face stimuli in ventral visual cortex can be seen as effects of perceptual expertise rather than of viewing faces *per se*. This builds on the notion that ventral visual cortex hosts brain areas that are not dedicated processors for various stimulus categories but instead areas of perceptual expertise^32, 33^. Similarly, electrophysiological work shows that perceptual familiarity with object images, gained over prolonged exposure, alters the response to objects in macaque IT^34^. In our experiment, participants completed a practice session prior to the behavioral and fMRI experiments and were shown a highly restricted set of four images during the task. This ensured familiarity with both the physical appearance as well as predictive relationships between our stimuli. Likewise, expertise for roman letters comes from a lifetime of reading and writing them in various fonts and sizes. While the stimuli used by Kovács and colleagues^31^ were likely recognizable for their participants, perceptual familiarity with the actual stimuli might have been lacking. It is therefore possible that perceptual familiarity, or access to highly detailed visual representations of the stimuli, facilitates expectation effects during visual processing.

### No effect of stimulus repetition in V1

Our study found no RS in V1. It is possible that this is a consequence of our task design: as there were only four stimuli in the experiment, these stimuli were shown very often throughout the experiment. This might have caused adaptation even for alternation trials, eventually abolishing RS in V1. We think this is unlikely however. First, this would beg the question why RS is absent in V1, but present in LOC. Second, studies on fMRI adaptation have shown that adaptation occurs even after many stimulus presentations^35^. Interestingly though, RS might depend on inter-stimulus interval (ISI) and occur only when the ISI is sufficiently short^36^. The same study also found that the ISI at which adaptation was found increased along the cortical hierarchy, consistent with the notion that temporal integration windows increase along the cortical hierarchy^37^. It is therefore possible that the ISI of our experiment exceeded the window during which V1 displays significant RS, but remained inside the window at which LOC exerts RS.

### Effects of stimulus expectation in V1

Do effects of stimulus expectation trickle down to primary visual cortex? So far, the evidence for this issue remains divergent: an initial investigation in which expectations were implemented as P_rep_ on faces revealed expectation effects in the lingual gyrus^38^. Yet a follow-up study, wherein stimulus expectation and repetition were varied independently, did not find any such back-propagation of stimulus expectations^9^ (while observing these at higher order areas). This led to the suggestion that stimulus expectation would generally not back-propagate to earliest visual areas when stimulus repetition and expectation were varied independently^9, 30^. Yet there are studies showing that the response of V1 is modulated by stimulus expectation to stimuli such as gratings^39–41^ or motion^42^ (see also ref. 43). Thus, it is an open question whether a stimulus expectation that acts on LOC (and RS in LOC) would also act on V1, especially since RS is in general much less pronounced in V1 than higher order areas^44, 45^.

In the current study we found that stimulus-specific voxels in the right primary visual cortex decreased their activity when stimuli were expected. This would suggest that stimulus expectations can indeed back-propagate to V1. This is in line with neurocomputational models that cast visual perception as a generative process. Here, expectations at higher levels of the cortical hierarchy propagate down to modulate sensory processing at the earlier levels, including V1^14–17^. Further work should be devoted to the complex interplay of visual areas during visual processing.

### Normal variation in autistic personality traits did not affect effects of expectation and repetition

Recent theoretical accounts cast autism as an altered balance between prior beliefs and sensory evidence^18–20^. Work by Ewbank and colleagues^21^ suggest that even normal variation in autistic personality traits predicts RS in ventral visual cortex (but not the effect of expectation on RS^22^). We did not observe such a modulation in our data, suggesting that the experimental effects observed in our specific paradigm are not modulated by normal variation in autistic personality traits.

## Conclusion

We found that repetition suppression (RS) to objects in LOC was modulated by stimulus-specific expectations, such that RS was more pronounced for unexpected relative to expected stimuli. Additionally, we found that the response of stimulus-preferring voxels in V1 was generally decreased when stimuli were expected. This pattern of results suggests that stimulus-specific expectations about objects modulate LOC and propagate back to the earliest cortical station processing visual input.

## Electronic supplementary material

Supplemental information
