# Supplementary material for: Repetition suppression to objects is modulated by stimulus-specific expectations
Source: Sci Rep. 2017 Aug 18;7:8781. doi: 10.1038/s41598-017-09374-z (PMC5562860; doi:10.1038/s41598-017-09374-z)
Supplement: Supplementary file 1 — Supplemental information [file 41598_2017_9374_MOESM1_ESM.pdf]

Supplemental information

Title:

Repetition suppression to objects is modulated by stimulus-specific expectations

Authors:

Christian Utzerath[1,2*], Elexa St. John-Saaltink[2], Jan Buitelaar[1,2] & Floris P. de Lange[2]

[1] Radboudumc, Donders Institute for Brain, Cognition, and Behaviour, Department of Cognitive Neuroscience, Geert Grooteplein-Noord 21, 6525 EZ Nijmegen, The Netherlands

[2] Radboud University, Donders Institute for Brain, Cognition and Behaviour
Kapittelweg 29, 6525 EN Nijmegen, The Netherlands

** Corresponding author*
Christian Utzerath
Radboudumc, Radboud University
Donders Institute for Brain, Cognition and Behaviour
6525 EN Nijmegen, The Netherlands
E-Mail: c.utzerath@donders.ru.nl
Phone: 0031 24 361 0363

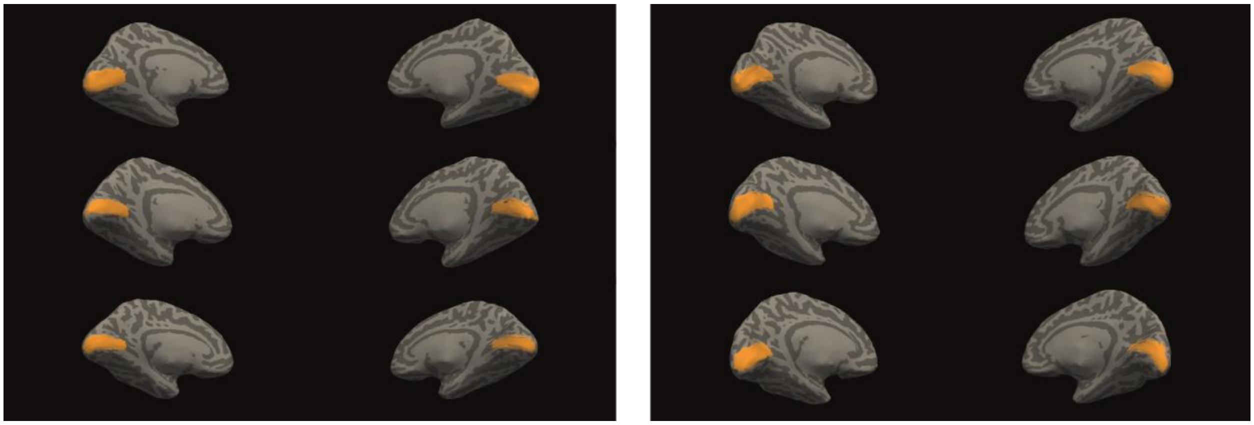

Figure S1 (relates to Figure 3). Anatomical reconstruction of V1. V1 was reconstructed using Freesurfer (https://surfer.nmr.mgh.harvard.edu/). Panels show the location of left and right V1 on the inflated cortical surface of five representative participants. The borders of V1 were predicted based on cortical folding, using automatic algorithms[1], which is possible with a high degree of accuracy[2]. Reconstructed anatomical scans were brought in register with each participant's functional scans, converted into a volume, and then finally transformed into MNI space using SPM8. This resulted in volume masks, from which voxels could be sampled for further analysis.

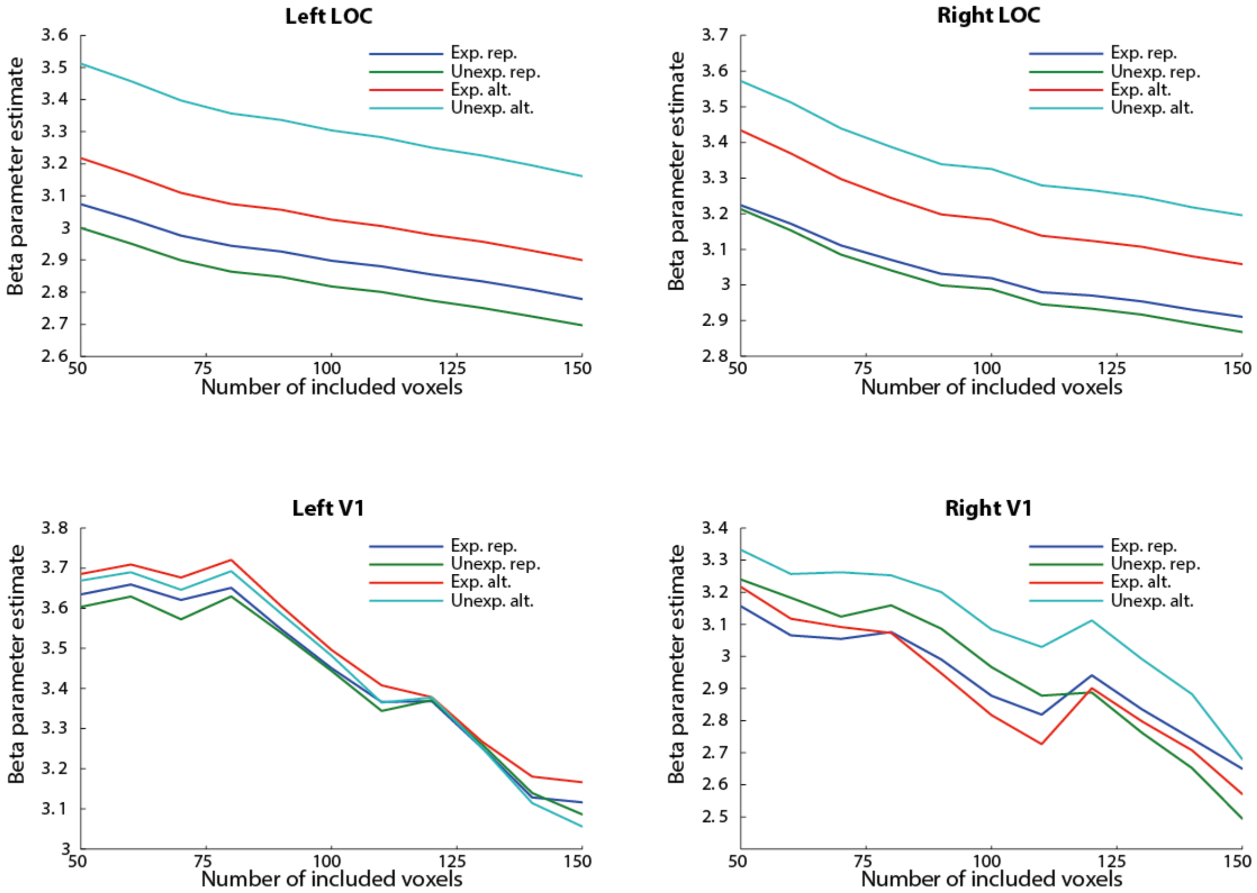

Figure S2. Consistent response of ROI as ROI sizes are increased. Beta parameters tend to decrease in every hemisphere and ROI as more voxels are included, preserving the relative difference between conditions. Although beta parameters are noisier in V1, the overall pattern of conditions is preserved across conditions. This suggests that the voxels selected in our ROI procedure are indeed sensitive for the task.

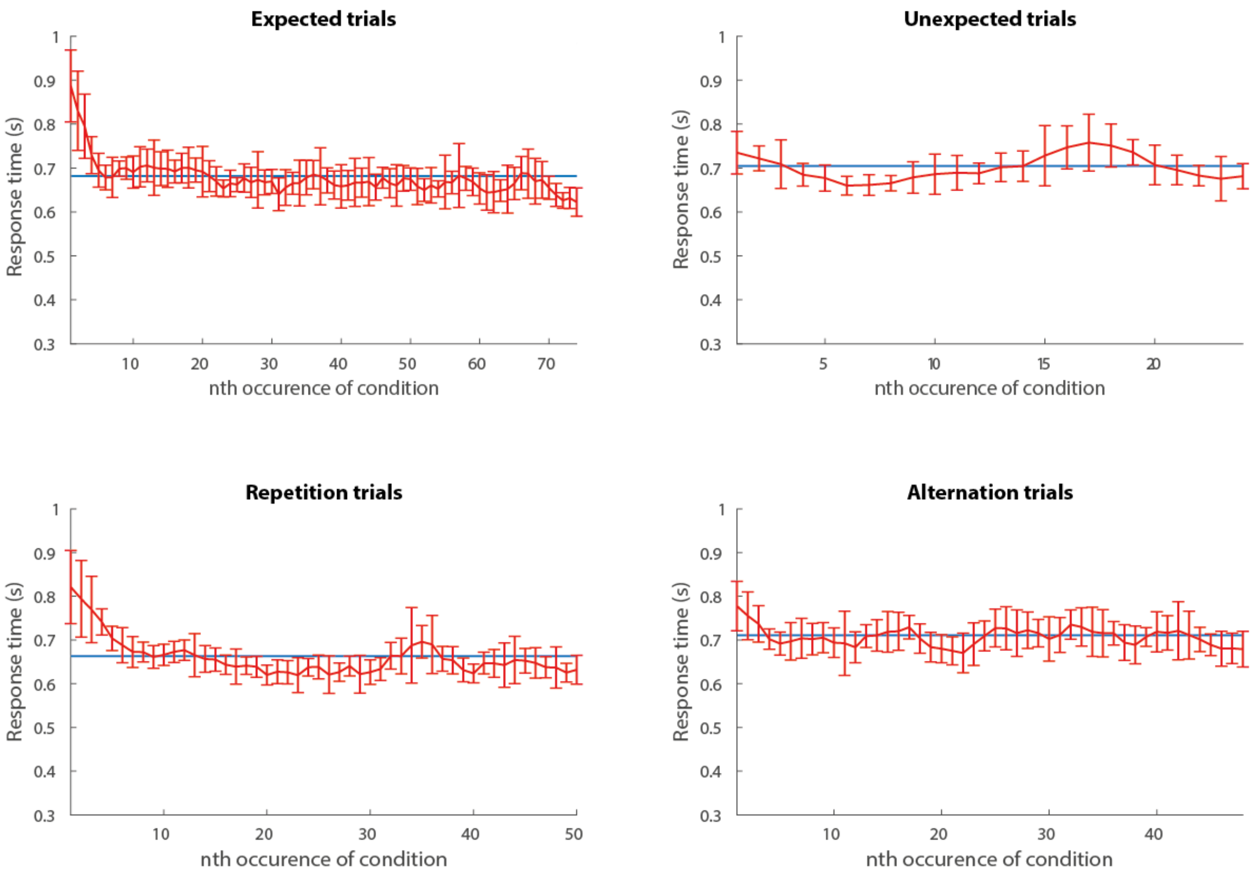

Figure S3. Moving average of response times during behavioral experiment suggest that participants had learned the stimulus transitions before entering the fMRI experiment. We calculated moving averages with a window size of three trials for each level of the factors expectation and repetition separately – that is, expected and unexpected trials, as well as repetitions and alternations. We chose these trial types as they correspond to the main effects of expectation and repetition we observed. The red line shows moving average reaction time per factor level. The blue lines correspond to each condition's mean reaction time. Error bars reflect within-trial standard error of the mean across the sample. This suggests that participants had learned the probabilistic structure of the experiment when they proceeded to the fMRI experiment.

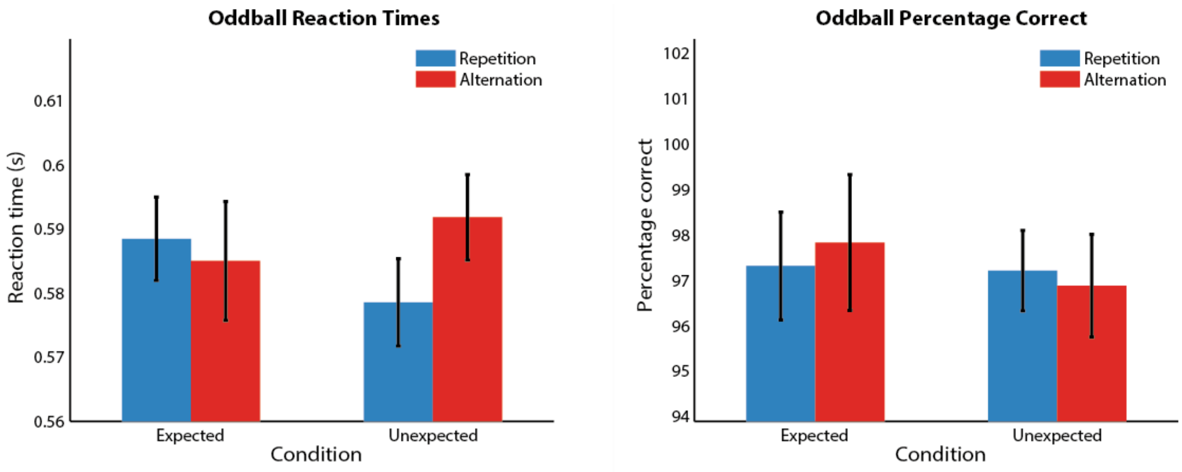

Figure S4. Reaction times and percentage correct from the oddball task during fMRI. There were no effects of repetition or expectation, nor did the two factors interact.

Development of expectation effects in LOC

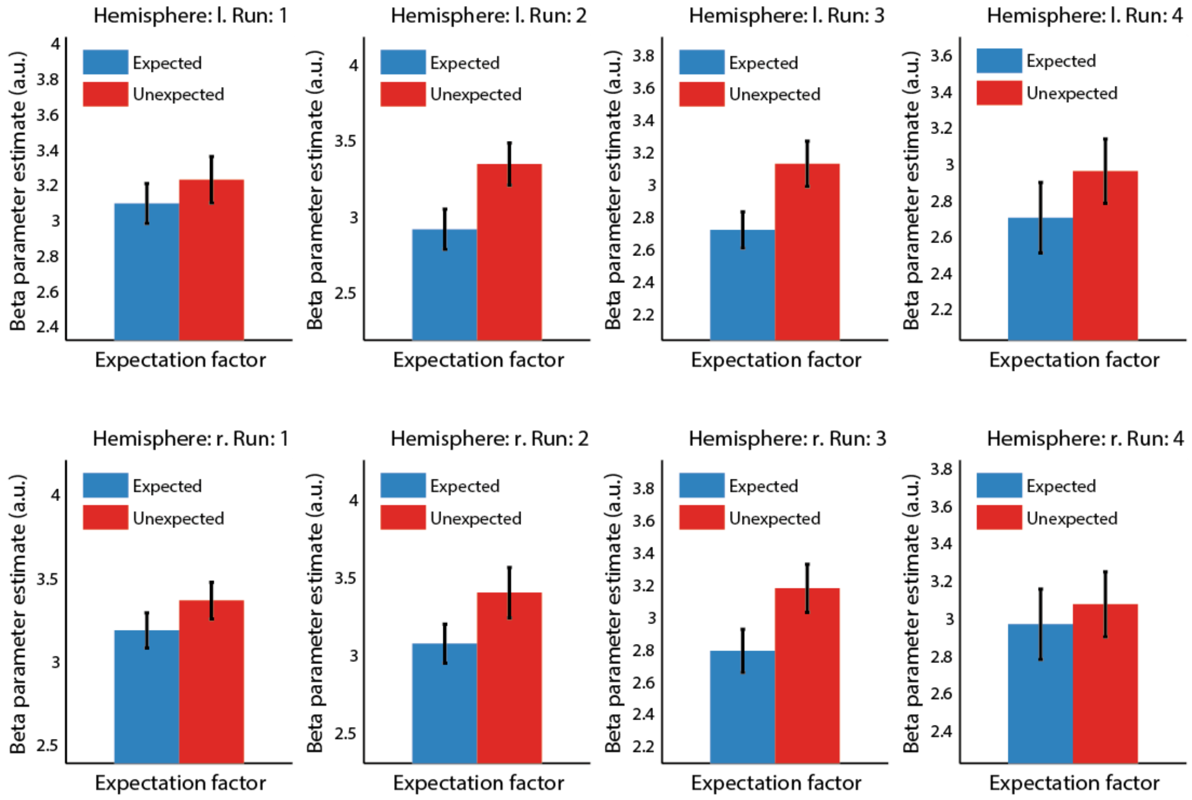

Development of expectation effects in V1

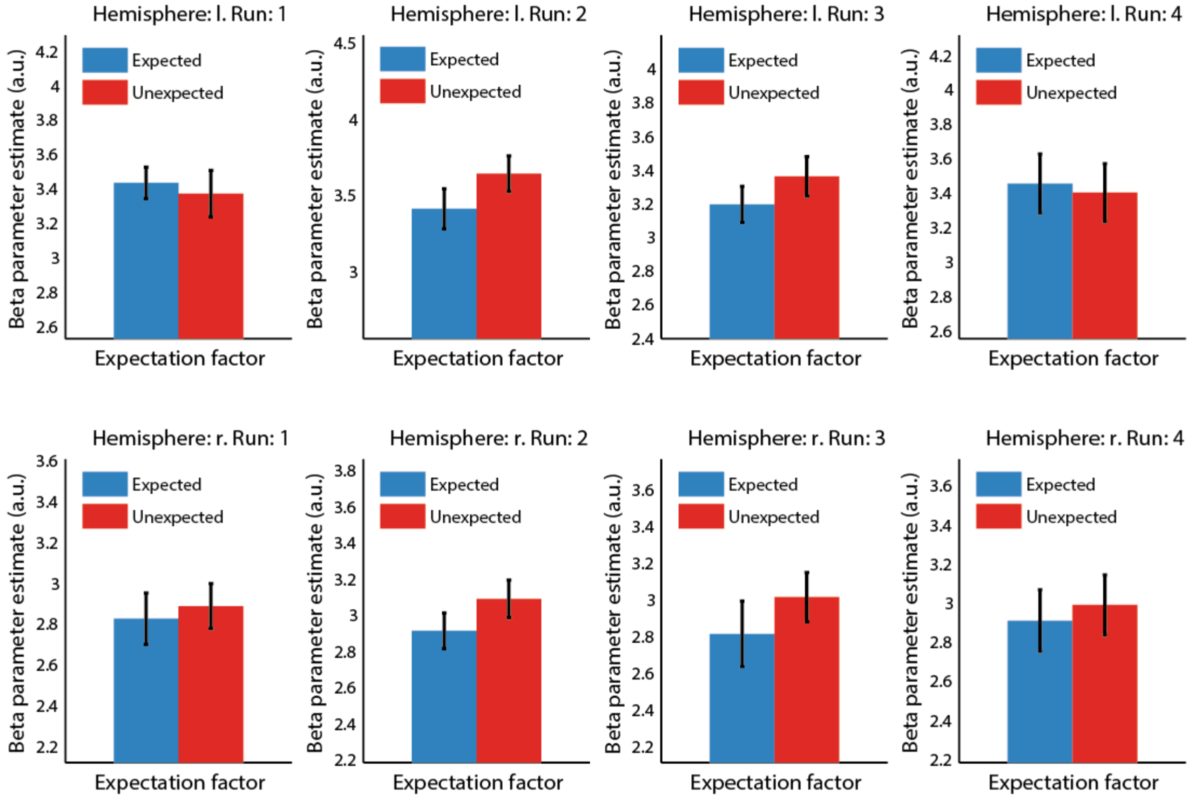

Figure S5. Development of expectation effects in LOC and V1. Top rows show left and right LOC, bottom rows show left and right V1. Bars represent mean beta parameter estimates for expected and unexpected trials. To test whether effects of expectation on the BOLD response are stable over time, we re-analyzed our ROI data with the factors ROI(V1, LOC) x Run(1,2,3,4) x Expectation(expected, unexpected) x Repetition (repetition, alternation) x Hemisphere(left, right). The expectation x repetition interaction was significant ($F(1,23) = 3.89$, $p = 0.0486$). Expectation did not interact with Run, Hemisphere, or ROI. Repetition on the other hand interacted with ROI significantly ($F(1,23) = 4.85$, $p = 0.0278$), as it was significantly present in LOC but not V1. Repetition did not interact with run or hemisphere. As for other effects, run, ROI, and hemisphere did not have significant main effects on beta amplitudes, although run and ROI interacted ($F(1,23) = 3.28$, $p = 0.0203$). This analysis suggests that expectation effects did not vary over time.

Supplementary references

1. Hinds, O. P. *et al.* Accurate prediction of V1 location from cortical folds in a surface coordinate system. *Neuroimage* **39,** 1585–1599 (2008).

2. Benson, N. C. *et al.* The Retinotopic Organization of Striate Cortex Is Well Predicted by Surface Topology. *Curr. Biol.* **22,** 2081–2085 (2012).
